# Optimizing patient selection for neoadjuvant immunotherapy in gastric cancer via pre-treatment imaging analysis

**DOI:** 10.3389/fimmu.2026.1848969

**Published:** 2026-08-05

**Authors:** Hao-Xiang Zhang, Ze-Ning Huang, Qiu-Xian Chen, Lei Wang, Feng Chen, Cai-Ming Weng, Yi-Fan Li, Yi Li, Zhi-Wei Liu, Jin-Quan Chen, Qi-Yue Chen, Jia-Bin Wang, Ping- Li, Chao-Hui Zheng, Chang-Ming Huang, Bin Wang, Li-Sheng Cai, Jian-Xian Lin, Jian-Wei Xie

**Affiliations:** 1 Department of Gastric Surgery, Fujian Medical University Union Hospital, Fuzhou, China; 2 Key Laboratory of Ministry of Education of Gastrointestinal Cancer, Fujian Medical University, Fuzhou, China; 3 Department of Gastrointestinal Surgery, Zhangzhou Affiliated Hospital of Fujian Medical University, Zhangzhou, China; 4 Department of Gastroenterology, Chongqing Key Laboratory of Digestive Malignancies, Daping Hospital, Army Medical University (Third Military Medical University), Chongqing, China

**Keywords:** gastric cancer, immunotherapy, major pathological response, neoadjuvant therapy, radiomics

## Abstract

**Background:**

The clinical efficacy of neoadjuvant immunotherapy combined with chemotherapy (NICT) in patients with locally advanced gastric cancer (LAGC) exhibits considerable variability. In this study, we developed and validated a radiomics-based scoring system to inform personalized neoadjuvant therapy strategies.

**Methods:**

A retrospective analysis was conducted on a multicenter cohort comprising 633 LAGC patients who underwent neoadjuvant therapy between 2019 and 2023. The primary endpoint was major pathological response (MPR). Radiomic features were extracted and selected from contrast-enhanced CT images obtained before neoadjuvant therapy to construct a radiomics scoring system, which stratified patients into high-, intermediate-, and low-score groups. The therapeutic outcomes across different groups were compared to discern patients who are likely to benefit from NICT as opposed to those who would derive more benefit from neoadjuvant chemotherapy alone (NACT). Additionally, analysis of tumor microenvironment immune infiltration and functional enrichment were performed to explore the potential biological mechanisms underlying the radiomic signatures.

**Results:**

In the intermediate-score group, the MPR rates were significantly higher among patients receiving NICT compared to those receiving NACT (training set: 49.2% vs. 26.0%, *p* = 0.005; validation set: 51.6% vs. 19.2%, *p* = 0.002). In contrast, no significant differences were observed in the high- or low-score groups. The analysis of immune infiltration indicated that the intermediate-score group exhibited significantly higher baseline levels of mast cells and dendritic cells compared to the combined high- and low-score groups (*p* < 0.05). Post-treatment evaluation following NICT revealed a significant increase in CD8+ T-cell infiltration relative to baseline levels within the intermediate-score group (*p* = 0.033). The gene KRT5 was identified as differentially expressed and correlated with dendritic cell abundance.

**Conclusion:**

The radiomics scoring system exhibits potential for stratifying patients who may be appropriate candidates for NICT or for whom the de-escalation of immunotherapy might be considered in future prospective studies, thereby contributing to the optimization of neoadjuvant treatment strategies.

## 1 Introduction

Gastric cancer (GC) ranks as the fifth most prevalent malignancy worldwide and constitutes the third leading cause of cancer-related mortality (1). Despite recent advancements in diagnostic methodologies and therapeutic interventions, survival rates remain suboptimal (2, 3). Neoadjuvant therapy (NAT), employed as a preoperative systemic treatment, has the potential to decrease tumor burden and enhance resectability, thereby improving patient prognosis (4, 5). Nevertheless, the variability in individual responses to neoadjuvant therapy underscores the necessity for a reliable predictive system to identify patients who are most likely to benefit from specific treatment regimens (6).

Traditionally, neoadjuvant therapy has predominantly utilized chemotherapy; however, the emergence of immunotherapy has opened new avenues for the treatment of gastric cancer (7, 8). Clinical trials have indicated that the combination of immunotherapy and chemotherapy can produce favorable outcomes in selected patient cohorts (9, 10). Despite its potential, immunotherapy does not universally yield positive outcomes and may induce adverse events of varying severity that can detrimentally affect patients’ quality of life and adherence to treatment regimens (11, 12). Consequently, a significant challenge in the management of gastric cancer is the precise identification of patients who will benefit from neoadjuvant immunotherapy. At present, the Combined Positive Score (CPS) is extensively employed to evaluate PD-L1 expression and guide the selection of patients for immunotherapy (13, 14). However, CPS relies on the immunohistochemical detection of PD-L1 expression in both tumor and immune cells, making it vulnerable to tissue sampling bias, variability in testing standards, and an inability to fully capture tumor heterogeneity (15–17). Clinical trials have demonstrated that even patients with low CPS may experience survival benefits from immunotherapy (18). Therefore, relying solely on the CPS may insufficiently identify patients who would truly benefit from immunotherapy.

Radiomics has been demonstrated to provide valuable insights for predicting treatment response in gastric cancer (19). By elucidating tumor heterogeneity, radiomics can offer potential biomarkers for therapeutic response (20). In recent years, radiomics has shown extensive applicability in diagnosis, efficacy assessment, and prognosis prediction across various malignancies (21, 22). In the context of gastric cancer, radiomic signatures have been employed to predict lymph node metastasis, postoperative recurrence risk, and to guide individualized treatment selection (23, 24). Notably, in the neoadjuvant setting, radiomics has proven effective in predicting treatment response, thereby aiding clinical decision-making (25, 26). Nevertheless, this approach provides limited utility in informing the crucial clinical decision regarding the incorporation of immunotherapy.

Building upon these insights, this study aims to develop a non-invasive, radiomics-based scoring system that utilizes pre-neoadjuvant CT imaging to identify patients likely to benefit from neoadjuvant immunotherapy. Furthermore, it aims to investigate candidates for potential immunotherapy de-escalation in future prospective studies. In addition to clinical decision support, the study emphasizes and explores potential associations between radiomic features and the tumor immune microenvironment.

## 2 Materials and methods

### 2.1 Study design and participants

**Figure 1:**
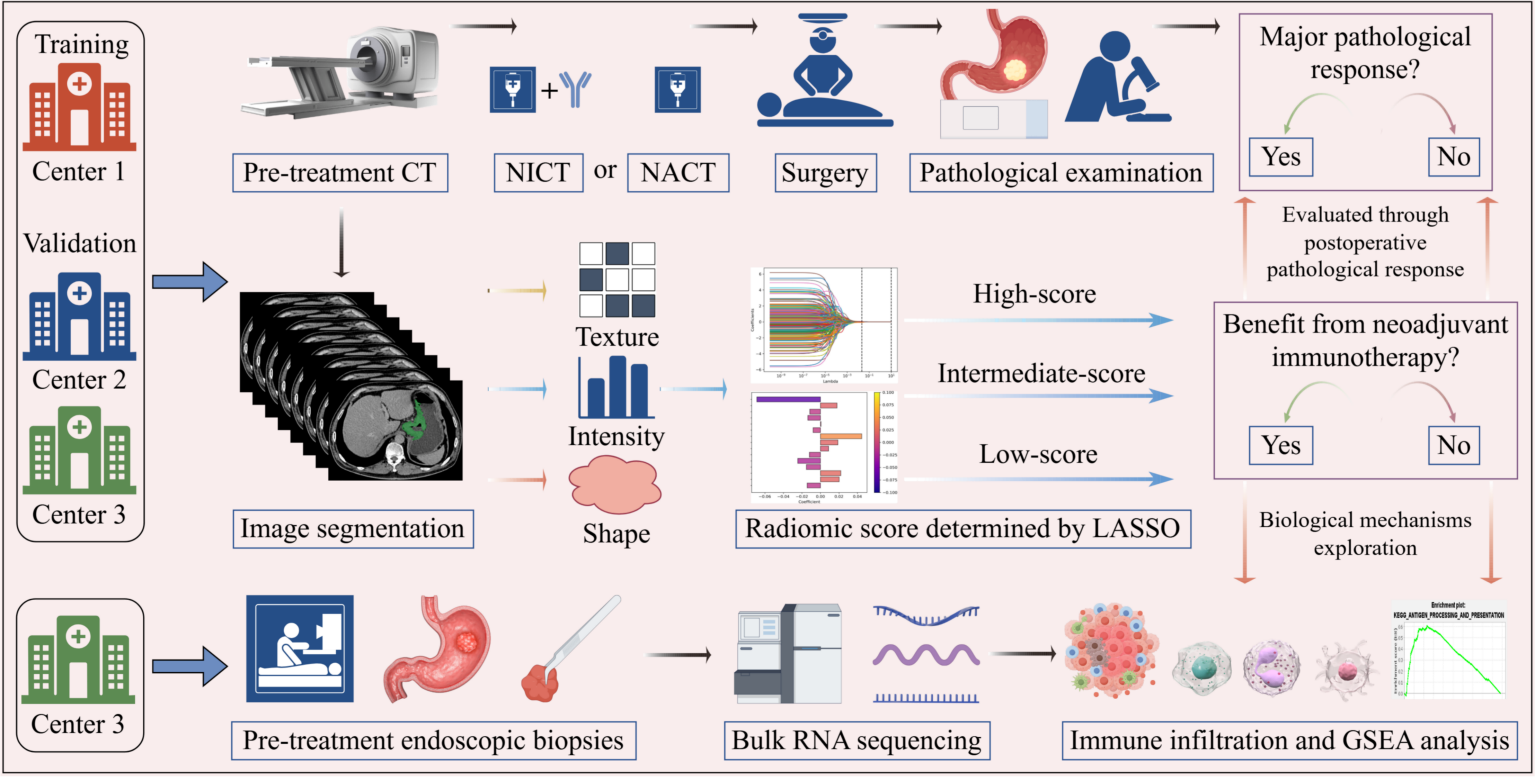
The process of developing and validating a radiomics score utilizing pre-neoadjuvant therapy imaging aims to predict pathological responses and guide personalized neoadjuvant therapy in locally advanced gastric cancer.

The overview of the study flow is presented in **Figure 1**. This retrospective, multicenter cohort study includes patients with locally advanced gastric cancer who underwent neoadjuvant therapy between January 2019 and December 2023 at Fujian Medical University Union Hospital (FJMUUH, Center 1), Zhangzhou Affiliated Hospital of Fujian Medical University (ZZMH, Center 2), and Daping Hospital of Army Medical University (DPH, Center 3). The inclusion criteria for the study were as follows: (1) histological confirmation of gastric adenocarcinoma via gastroscopy and biopsy; (2) patients with clinical stage cT3/T4N0/+M0 who had received preoperative neoadjuvant therapy; and (3) underwent radical gastrectomy. The exclusion criteria included: (1) receipt of treatment modalities other than neoadjuvant chemotherapy or immunochemotherapy, such as radiotherapy or targeted therapy; (2) presence of remnant gastric cancer; and (3) lack of pre-treatment CT images or poor image quality that impeded accurate extraction of radiomic features. In total, 414 patients from FJMUUH were allocated to the training set, while 219 patients from ZZMH and DPH constituted the external validation set (**Supplementary Figure 1**).

This study received approval from the Research Ethics Committee of FJMUUH. The Committee confirmed that the study protocol, the request for a waiver of informed consent, and all related documentation adhere to China’s Ethical Review Measures for Life Science and Medical Research Involving Humans and the Declaration of Helsinki (Ethics Approval No. 2025KY708). Due to the retrospective nature of the study and the lack of additional interventions affecting patient privacy, the Committee granted a waiver of individual informed consent. All human tissue samples utilized in this study were acquired and processed in full compliance with the World Health Organization Guiding Principles on Human Cell, Tissue, and Organ Transplantation. This study adhered to the Strengthening the Reporting of Observational Studies in Epidemiology (STROBE) reporting guidelines.

### 2.2 Study endpoint and patient characteristics

The primary endpoint of this study was the major pathological response rate (MPR), defined as the proportion of patients exhibiting less than 10% residual viable tumor cells in the resected surgical specimens. To evaluate the prognostic significance of MPR, Kaplan–Meier analyses of overall survival (OS) and disease-free survival (DFS) were conducted independently in both the training and validation sets. The clinical baseline characteristics were retrospectively gathered, encompassing both demographic and disease-specific parameters: age, sex, body mass index (BMI), tumor location, clinical T stage (cT), clinical N stage (cN), the number of neoadjuvant therapy cycles, and the treatment regimen. Additionally, postoperative pathological data were collected, which included the pathological T stage (ypT), pathological N stage (ypN), perineural invasion status, lymphovascular invasion status, tumor regression grade (TRG), and information regarding the administration of postoperative adjuvant therapy.

The neoadjuvant chemotherapy regimens implemented across the participating centers adhered rigorously to contemporary clinical guidelines. These regimens predominantly consisted of fluoropyrimidine- and platinum-based doublet combinations, such as SOX (S-1 plus oxaliplatin) and XELOX (capecitabine plus oxaliplatin), as well as triplet regimens incorporating taxanes, including FLOT (5-fluorouracil, leucovorin, oxaliplatin, and docetaxel) and DOS (docetaxel, oxaliplatin, and S-1). All regimens were consistent with the Chinese clinical practice guidelines and are routinely utilized in clinical settings. The immune checkpoint inhibitors employed in this study included sintilimab, nivolumab, and camrelizumab, which are PD-1/PD-L1 inhibitors approved by the National Medical Products Administration for the treatment of gastrointestinal malignancies, and these agents exhibit consistent mechanisms of action and clinical efficacy. The dosage and administration schedules for all therapeutic agents were meticulously followed as per the respective product labeling and guideline recommendations, with individualized adjustments made based on patient body surface area.

### 2.3 Image acquisition and processing

Within this study, all enrolled patients underwent contrast-enhanced abdominal CT scans prior to the initiation of neoadjuvant therapy. Imaging was conducted utilizing equipment from various manufacturers, including SIEMENS, GE Healthcare, and Philips. Despite this diversity, key acquisition parameters were maintained with remarkable consistency: the tube voltage was uniformly set at 120 kV; reconstruction kernels were consistently “STANDARD” or equivalent smooth kernels; and the slice thickness was fixed at 1.25 mm. A detailed summary of the acquisition parameters is available in **Supplementary Table 1**. The CT images were acquired from the portal venous phase axial series and retrieved from the Picture Archiving and Communication System (PACS) at each participating institution.

To ensure uniformity in image quality and analytical reproducibility, raw CT images underwent a standardized preprocessing pipeline, which included window-level adjustment, denoising, and isotropic resampling. Window-level adjustment was employed to optimize tumor visualization and minimize variations in brightness and contrast due to differences in imaging equipment or acquisition protocols, thereby ensuring a consistent image appearance. Denoising was executed using a Gaussian filter to suppress image noise and enhance clarity and interpretability. Finally, images were resampled to a uniform voxel size of 1*1*1 mm³ to achieve isotropic spatial resolution. This standardized workflow produced a homogeneous, quality-controlled dataset appropriate for robust radiomic feature extraction.

### 2.4 Radiomic feature extraction and selection

For this study, 3D Slicer (version 4.10.2) was utilized to manually delineate the largest axial cross-section of the tumor on CT images, with semi-automatic segmentation tools aiding in the definition of the region of interest. All annotations were independently conducted by an experienced radiologist. To evaluate reproducibility, the same radiologist re-segmented 50 randomly selected cases one month later, in collaboration with a second physician, who possesses a decade of experience in abdominal CT interpretation. A total of 832 radiomic features were extracted from the segmented volumes utilizing the PyRadiomics package (version 2.12), encompassing first-order statistics, texture features, and wavelet transformations. The feature extraction process adhered strictly to the Image Biomarker Standardization Initiative (IBSI) guidelines, thereby ensuring standardization and reproducibility.

The stability of these features was evaluated using the intra- and inter-class correlation coefficients (ICCs). ICCs < 0.5 indicate poor reliability, 0.5 to 0.75 indicate moderate reliability, 0.75 to 0.9 indicate good reliability, and > 0.9 indicates excellent reliability. Only features exhibiting ICCs of > 0.9 were retained for further analysis. All radiomic features were standardized using Z-score transformation based on the training set, which eliminated disparities in scale and units and ensured modeling stability and comparability. To refine the feature set, the least absolute shrinkage and selection operator (LASSO) regression with L1 regularization was employed within a 10-fold cross-validation framework, culminating in the identification of 15 non-zero coefficient features as key predictors. Utilizing these features and their respective LASSO coefficients (β_i_), a radiomic score (Radscore) was computed as Radscore = $\sum_{i=1}^{n}$ Radiomic_feature_i* βi), where β_i_ represents the regression coefficient of the i-th feature and Radiomic_feature_i_ denotes its corresponding value. Patients were stratified into low, intermediate, and high score groups according to the tertiles of the Radscore distribution.

### 2.5 Immune infiltration analysis

Pre-treatment endoscopic biopsies and post-resection specimens from 32 patients at Daping Hospital, who underwent NICT, were collected for bulk RNA sequencing. To quantify the immune cell composition across treatment phases, the CIBERSORT algorithm was employed. This algorithm utilizes linear support vector regression against a signature matrix of 22 immune cell phenotypes to deconvolute bulk gene expression data, thereby providing precise estimates of the relative abundance of immune cells (27). By aligning sample-specific gene expression profiles with predefined immune cell gene signatures, the algorithm facilitates high-resolution quantification of infiltrating immune populations within complex tissue environments. This methodology enhances analytical accuracy and deepens the characterization of the tumor immune microenvironment. Differences in immune infiltration between groups were assessed using the Wilcoxon rank-sum test, with *p*-values adjusted for multiple comparisons to control the false discovery rate.

### 2.6 Differential gene screening and network construction

To explore the molecular-level biological distinctions among the radiomic score groups, a differential expression analysis was conducted on the transcriptomic data of each group to identify genes exhibiting significant alterations. Differentially expressed genes (DEGs) were characterized by a |log_2_ fold change| greater than 2 and an adjusted *p*-value (*p*_adj) less than 0.05. To further elucidate the functional relationships among these DEGs, a protein-protein interaction network was constructed utilizing the STRING database (Search Tool for the Retrieval of Interacting Genes), with an interaction confidence score exceeding 0.7 to identify high-confidence hub genes. Subsequently, potential associations between the expression levels of these hub genes and the degree of immune cell infiltration were investigated. Pearson correlation analysis was employed to assess the relationships between gene expression and the abundance of various immune cell types, with *p*-values adjusted for multiple comparisons. These analyses contribute to a deeper understanding of the molecular connections between radiomic features and the tumor immune microenvironment, offering insights into the underlying biological mechanisms.

### 2.7 Pathway enrichment analysis

To elucidate the biological implications of the differentially expressed genes associated with gastric cancer, pathway enrichment analysis was conducted utilizing the TCGA-STAD (The Cancer Genome Atlas Stomach Adenocarcinoma) dataset. Gene Set Enrichment Analysis (GSEA) was employed to systematically evaluate the enrichment of specific genes within various biological pathways. This analysis incorporated canonical pathways from the Kyoto Encyclopedia of Genes and Genomes (KEGG) database, facilitating the comparison of gene expression profiles across distinct expression states. The GSEA software was utilized, with statistical significance established at *p* < 0.05, and *p*-values were adjusted for multiple comparisons to control the false discovery rate. Particular attention was given to signaling pathways related to immune response and tumor microenvironment regulation. The objective was to elucidate the potential functional roles of differentially expressed genes in immune modulation, thereby providing a theoretical framework for understanding the mechanisms underlying disease progression and therapeutic response.

### 2.8 Statistical analysis

The log-rank test was employed to compare the survival curves between the MPR and non-MPR groups. The discriminatory performance of the radiomic score in predicting MPR was assessed using receiver operating characteristic (ROC) curves and the area under the curve (AUC) for both the training and validation datasets. Calibration curves were employed to evaluate the concordance between predicted probabilities and observed outcomes, while decision curve analysis (DCA) was utilized to assess clinical utility. Univariable and multivariable logistic regression analyses were performed to identify independent predictors of MPR and to examine their associations across different strata of radiomic scores. Pearson correlation analysis was conducted to quantify linear relationships between radiomic features and gene expression levels or immune cell infiltration. Continuous variables are reported as mean ± standard deviation or median (interquartile range), and categorical variables as frequency (percentage). All statistical analyses were executed using R software (version 4.4.3), with a two-sided *p*-value < 0.05 considered indicative of statistical significance.

## 3 Results

### 3.1 Patient cohort and baseline characteristics

The proportion of patients aged over 65 years was 46.4% in the training set and 47.0% in the validation set, with no statistically significant difference (*p* = 0.941). However, there was a significant difference in the proportion of male patients between the two sets, with 79.7% in the training set compared to 68.9% in the validation set (*p* = 0.004). Tumors located in the middle third of the stomach were more prevalent in the validation set (29.7%) than in the training set (17.1%, *p* < 0.001). Additionally, a higher proportion of patients in the validation set received 3–4 cycles of neoadjuvant therapy (90.9%) compared to those in the training set (76.3%, *p* < 0.001). The proportion of patients with cN+ disease was lower in the validation set (88.6%) than in the training set (96.6%, *p* < 0.001). No significant differences were identified between the two groups in terms of the Type of neoadjuvant therapy or the incidence of postoperative adjuvant chemotherapy (all *p* > 0.05) (**Table 1**).

**Table 1:** Baseline, surgical, and pathological information of the patients.

Characteristic	Subgroup	Training cohort (n = 414)	Testing cohort (n = 219)	*P* value
Age (%)	≥65	192 (46.4)	103 (47.0)	0.941
	<65	222 (53.6)	116 (53.0)	
Sex (%)	Female	84 (20.3)	68 (31.1)	0.004
	Male	330 (79.7)	151 (68.9)	
BMI (%)	≥25	86 (20.8)	23 (10.5)	0.002
	<25	328 (79.2)	196 (89.5)	
Tumor Location (%)	Upper	169 (40.8)	94 (42.9)	<0.001
	Middle	71 (17.1)	65 (29.7)	
	Lower	99 (23.9)	56 (25.6)	
	Mix	75 (18.1)	4 ( 1.8)	
Clinical T stage (%)	T3	63 (15.2)	58 (26.5)	0.001
	T4	351 (84.8)	161 (73.5)	
Clinical N stage (%)	N0	14 ( 3.4)	25 (11.4)	<0.001
	N+	400 (96.6)	194 (88.6)	
Cycle of NAT (%)	1-2	36 ( 8.7)	9 ( 4.1)	<0.001
	3-4	316 (76.3)	199 (90.9)	
	≥5	62 (15.0)	11 ( 5.0)	
Type of NAT (%)	NACT	244 (58.9)	131 (59.8)	0.897
	NICT	170 (41.1)	88 (40.2)	
ypT category (%)	pCR	60 (14.5)	24 (11.0)	<0.001
	T1	44 (10.6)	21 ( 9.6)	
	T2	44 (10.6)	21 ( 9.6)	
	T3	192 (46.4)	42 (19.2)	
	T4	74 (17.9)	111 (50.7)	
ypN category (%)	N0	192 (46.4)	76 (34.7)	0.016
	N1	84 (20.3)	51 (23.3)	
	N2	53 (12.8)	44 (20.1)	
	N3	85 (20.5)	48 (21.9)	
Perineural invasion (%)	Absent	216 (52.2)	80 (36.5)	<0.001
	Present	198 (47.8)	139 (63.5)	
Lymphovascular invasion (%)	Absent	255 (61.6)	99 (45.2)	<0.001
	Present	159 (38.4)	120 (54.8)	
TRG (%)	1a	60 (14.5)	23 (10.5)	0.064
	1b	82 (19.8)	42 (19.2)	
	2	132 (31.9)	92 (42.0)	
	3	140 (33.8)	62 (28.3)	
Adjuvant chemotherapy (%)	No	35 ( 8.5)	28 (12.8)	0.111
	Yes	379 (91.5)	191 (87.2)	

BMI, body mass index; NAT, neoadjuvant therapy; NACT, neoadjuvant chemotherapy; NICT, neoadjuvant immunochemotherapy; TRG, tumor regression grade.

### 3.2 MPR is significantly associated with long-term survival outcomes

In both the training and validation sets, patients achieving MPR exhibited significantly improved 2-year OS and DFS rates relative to non-MPR patients (Training: OS, 92.3% vs. 70.6%, and DFS, 87.3% vs. 62.1%; Validation: OS, 86.2% vs. 66.2%, and DFS, 84.6% vs. 56.5%, respectively; all *p* < 0.01) (**Supplementary Figure 2**).

### 3.3 Development and predictive performance of the radiomic score

A total of 832 radiomic features were extracted from baseline CT-defined tumor regions of interest, encompassing attributes such as tumor size, morphology, density, and texture. Following the evaluation of the ICC, 606 features demonstrating high stability (ICC > 0.9) were retained (**Supplementary Figure 3**). Utilizing the LASSO regression with 10-fold cross-validation, 15 features with the highest predictive value were selected from these stable variables and combined linearly to construct the radiomic score (Radscore = $\sum_{i=1}^{n}$ Radiomic_feature_i* βi) (**Supplementary Figure 4**). Patients were stratified into high-score (> 0.3946), intermediate-score (0.3089-0.3946), and low-score (< 0.3089) groups based on the cutoff values derived from the Radscore distribution (**Supplementary Figure 5**).

**Figure 2:**
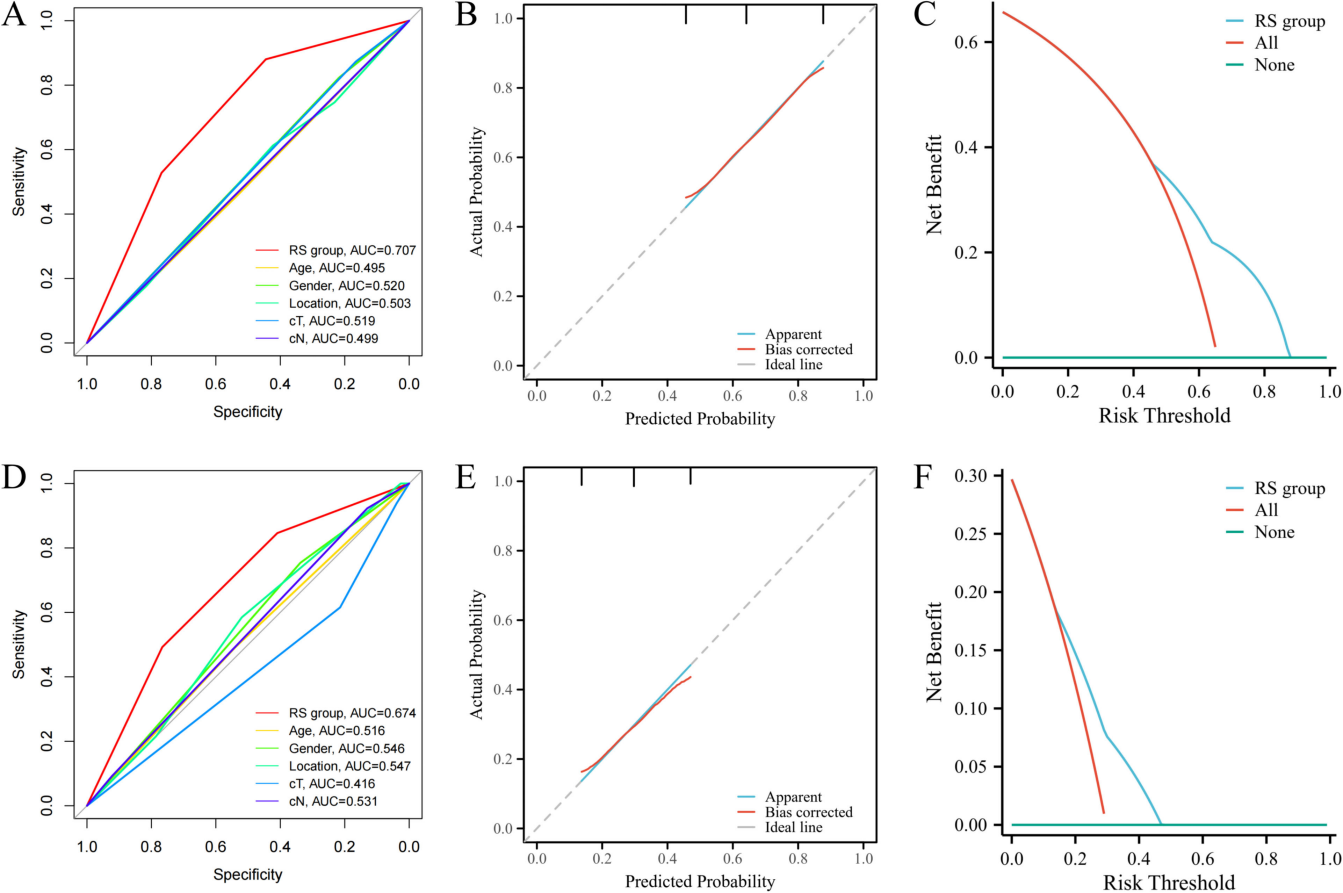
Discrimination, calibration, and clinical utility of radscore-based grouping for predicting major pathological response in both the training and validation sets, featuring: **(A)** the ROC curve for the training set, **(B)** the calibration curve for the training set, **(C)** the clinical decision curve for the training set, **(D)** the ROC curve for the validation set, **(E)** the calibration curve for the validation set and **(F)** the clinical decision curve for the validation set.

In the training dataset, the radiomic score achieved an AUC of 0.707 in predicting MPR, surpassing other factors such as age, sex, tumor location, clinical T stage, and clinical N stage (**Figure 2A**). Calibration curves indicated a strong concordance between predicted probabilities and observed outcomes (**Figure 2B**), while decision curve analysis revealed a significant net clinical benefit across a range of risk thresholds (**Figure 2C**). In the independent validation dataset, the radiomic score attained an AUC of 0.674, demonstrating satisfactory calibration and decision curve performance (**Figures 2D–F**), suggesting the model’s potential utility and generalizability.

### 3.4 Comparison of MPR rates across radiomic score groups and univariable/multivariable analysis

**Figure 3:**
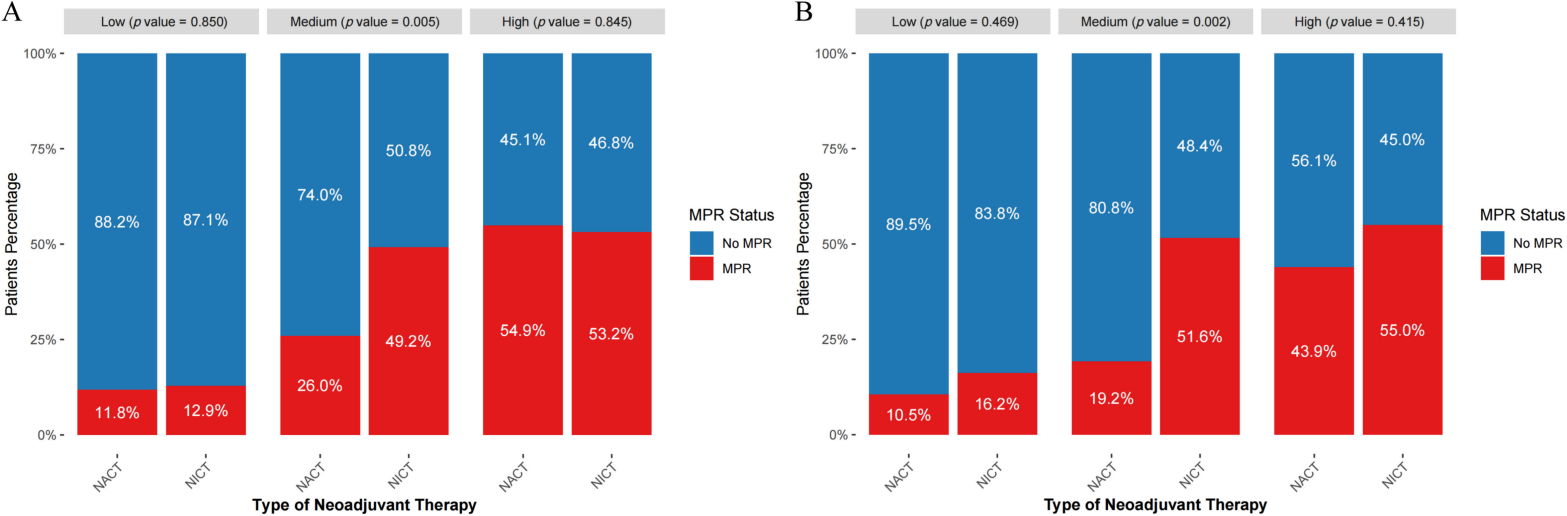
A comparative analysis of major pathologic response rates for various neoadjuvant therapies across the low-score, intermediate-score, and high-score groups in both **(A)** the training set and **(B)** the validation set.

Within the training set, there was no statistically significant difference in MPR rate between NICT and NACT in either the high-score group (NICT 53.2% vs. NACT 54.9%, *p* = 0.845) or the low-score group (NICT 12.9% vs. NACT 11.8%, *p* = 0.850). Notably, within the intermediate-score group, NICT yielded a significantly higher MPR rate compared to NACT (49.2% vs. 26.0%, *p* = 0.005), indicating a substantial advantage conferred by immunotherapy (**Figure 3A**). This trend was corroborated in the validation set, with the high-score group showing MPR rates of 55.0% versus 43.9% (*p* = 0.415), the low-score group 16.2% versus 10.5% (*p* = 0.469), and the intermediate-score group 51.6% versus 19.2% (*p* = 0.002) (**Figure 3B**).

Univariable and multivariable logistic regression analysis, adjusted for age, sex, tumor location, clinical T stage, clinical N stage and chemotherapy regimen, revealed that NICT was not an independent protective factor in the combined high- and low-score groups (odds ratio [OR] = 0.59, *p* = 0.084) (**Supplementary Table 2**), whereas it was identified as an independent protective factor in the intermediate-score group (OR = 4.34, *p* = 0.001) (**Supplementary Table 3**). The findings were consistent with those observed in the validation set (**Supplementary Tables 4**, **5**).

### 3.5 Clinical characteristics, PD-L1 expression, and immune infiltration across radiomic score groups

Baseline clinical variables in the intermediate-score group were compared with those in the combined high- and low-score groups within the training set. No significant differences were observed among the three groups concerning age, sex, BMI, tumor location, or clinical T/N stage (**Supplementary Figure 6**). Furthermore, PD-L1 expression levels were similar across the three groups (**Supplementary Figure 7**), suggesting that the radiomic score captures treatment responsiveness through mechanisms independent of PD-L1 status.

**Figure 4:**
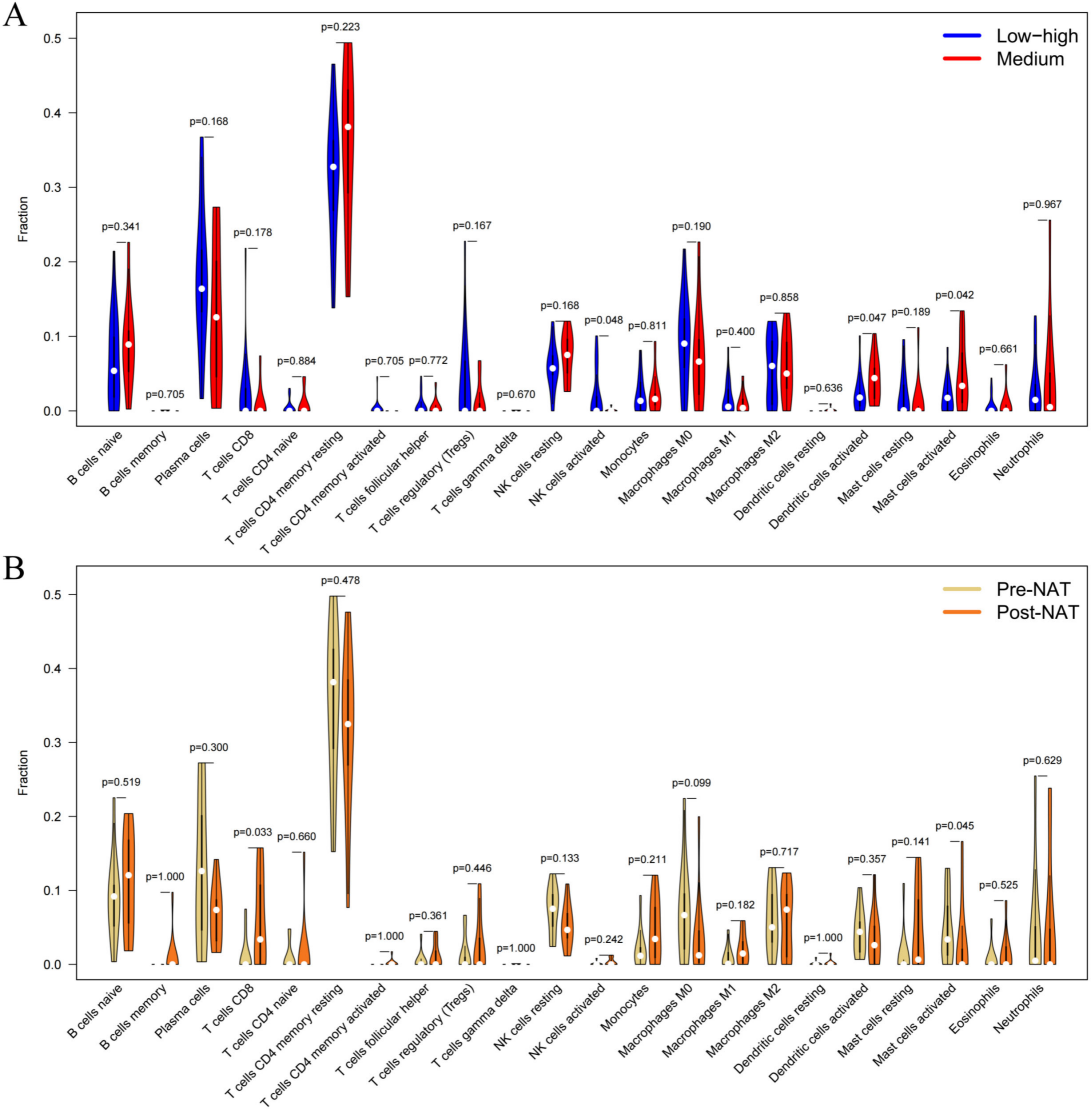
**(A)** A bar plot depicting immune cell expression in the intermediate-score group versus the combined high- and low-score groups, and **(B)** a bar plot illustrating changes in immune cell expression before and after neoadjuvant therapy (pre-NICT vs. post-NICT) within the intermediate-score group.

The analysis of pre-treatment immune infiltration demonstrated that tumors classified within the intermediate-score group exhibited significantly elevated levels of activated mast cells (*p* = 0.042) and activated dendritic cells (*p* = 0.047) in comparison to those in the combined high- and low-score groups (**Figure 4A**). Furthermore, within the intermediate-score subgroup, the comparison of immune profiles before and after treatment revealed a significant increase in CD8^+^ T-cell abundance post-treatment (*p* = 0.033) (**Figure 4B**), indicating the effective induction of antigen-specific cytotoxic T-cell responses by NICT.

### 3.6 Differential expression analysis and correlation between the candidate gene and immune infiltration

**Figure 5:**
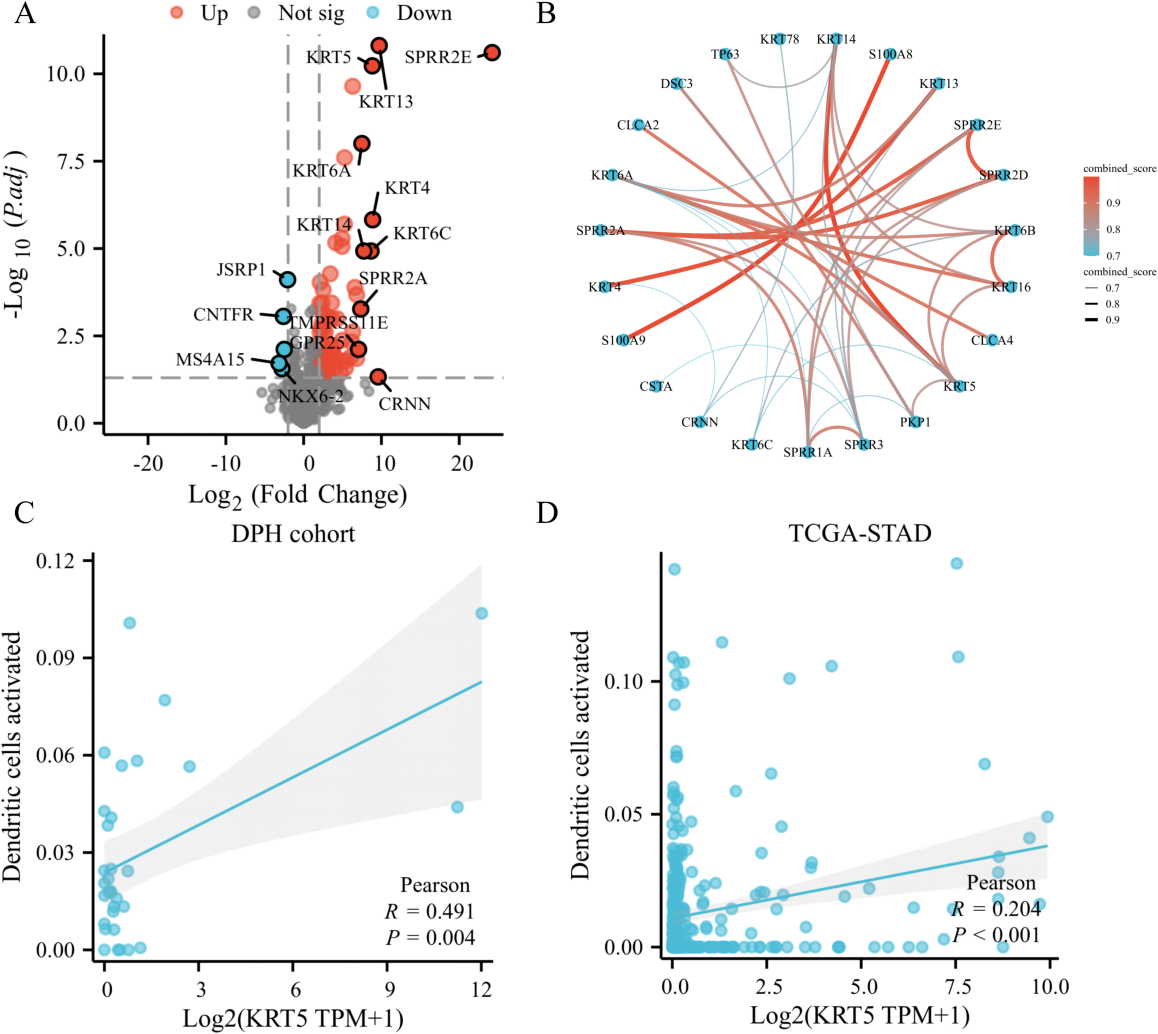
**(A)** A volcano plot illustrating the differentially expressed genes between the intermediate-score group and the combined high- and low-score groups, **(B)** a protein-protein interaction network of the identified differentially expressed genes, **(C)** a Pearson correlation plot demonstrating the relationship between the expression level of the core gene KRT5 and dendritic cell infiltration in the DPH cohort, and **(D)** the same correlation in the TCGA-STAD dataset.

By applying thresholds of |log_2_FC| > 2 and an adjusted *p*-value (*p*_adj) < 0.05, 75 significantly differentially expressed genes were identified, including 70 upregulated and 5 downregulated genes (**Figure 5A**). A protein-protein interaction network was constructed using the STRING database, with an interaction confidence score greater than 0.7 to ensure reliability. Within this network, KRT5 (keratin 5) emerged as a central hub gene with high connectivity, indicating a potential regulatory role in the associated functional gene module and positioning KRT5 as a candidate for further functional investigation (**Figure 5B**). **Supplementary Figure 8** highlights KRT5 and elucidates its direct and indirect interactions with other genes.

Our analysis revealed a statistically significant positive correlation between KRT5 expression and the abundance of activated dendritic cells, with a correlation coefficient of R = 0.491 (*p* = 0.004). This relationship was independently corroborated in the TCGA-STAD set, where KRT5 expression similarly exhibited a positive correlation with the infiltration of activated dendritic cells, yielding a correlation coefficient of R = 0.204 (*p* < 0.001) (**Figures 5C, D**).

### 3.7 Pathway enrichment analysis

**Figure 6:**
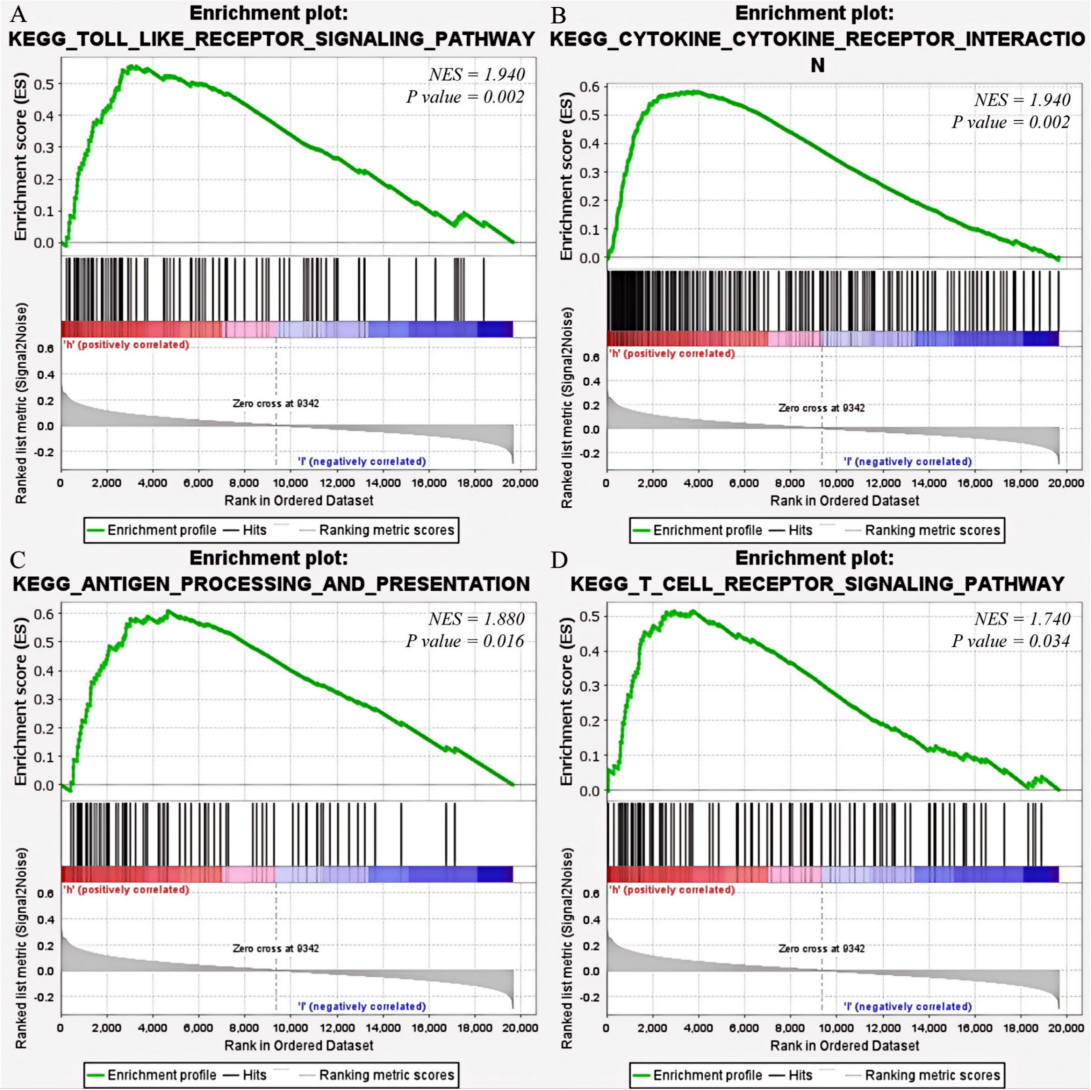
Gene Set Enrichment analysis of KEGG pathways associated with varying KRT5 expression levels (high vs. low) in the TCGA-STAD dataset: **(A)** toll-like receptor signaling, **(B)** cytokine-cytokine receptor interaction, **(C)** antigen processing and presentation, and **(D)** T cell receptor signaling.

A systematic comparison of gene expression profiles between KRT5-high and KRT5-low groups revealed significant enrichment of several key immune-related pathways in the KRT5-high group, including KEGG_TOLL_LIKE_RECEPTOR_SIGNALING_PATHWAY, KEGG_CYTOKINE_CYTOKINE_RECEPTOR_INTERACTION, KEGG_ANTIGEN_PROCESSING_AND_PRESENTATION, and KEGG_T_CELL_RECEPTOR_SIGNALING_PATHWAY (**Figure 6**).

### 3.8 Correlation analysis and potential biological associations

Correlation analysis indicated moderate positive associations between KRT5 expression and specific radiomic features—wavelet.LHH_glrlm_ShortRunLowGrayLevelEmphasis, wavelet.LHH_glszm_LowGrayLevelZoneEmphasis, wavelet.HHL_glszm_LargeAreaLowGrayLevelEmphasis, and wavelet.LHL_glcm_Imc1—with Pearson correlation coefficients ranging from 0.25 to 0.5. In a similar vein, the infiltration level of activated dendritic cells exhibited moderate positive correlations with two specific features—wavelet.LHH_glrlm_ShortRunLowGrayLevelEmphasis and wavelet.LHH_glszm_LowGrayLevelZoneEmphasis—with Pearson correlation coefficients ranging from 0.25 to 0.5 (**Supplementary Figure 9**).

## 4 Discussion

This study developed a radiomics-based scoring system to enhance patient selection for neoadjuvant therapy in locally advanced gastric cancer, with the goal of improving therapeutic efficacy while reducing unnecessary treatment-related risks. The radiomic score exhibited potential discriminative capability for predicting major pathological response, thereby facilitating the identification of patient subgroups with diverse treatment outcomes. This performance was consistent across both the training and validation datasets. The results of this study highlight the potential utility of the radiomic score as an imaging biomarker for stratifying patients who may be most likely to benefit from neoadjuvant immunotherapy, while also suggesting those who may achieve satisfactory outcomes with chemotherapy alone. This methodology has the potential to mitigate immune-related toxicities and enhance the overall therapeutic efficacy. The proposed scoring framework is anticipated to be a valuable asset in guiding personalized treatment strategies for gastric cancer.

The radiomic score was derived from an extensive array of highly stable radiomic features that thoroughly quantify tumor volume, shape, density, and texture, thereby encapsulating the full spectrum of tumor heterogeneity. The key imaging variables identified by the LASSO regression model highlight the promising role of radiomics in oncological diagnosis and the prediction of treatment responses. Compared to traditional clinical parameters, the radiomic score demonstrated additional discriminatory capability in predicting major pathological response, indicating its potential to capture underlying biological characteristics not fully represented by conventional indices. Our findings suggest a potential association between certain radiomic features and KRT5 expression, as well as the presence of activated dendritic cells within the tumor microenvironment. This suggests that radiomic signatures may reflect underlying molecular and immunological heterogeneity in tumor tissues. We acknowledge that stratifying patients by tertiles involves inherent limitations related to cut-off selection. Nonetheless, this stratification method was meticulously designed to balance statistical distribution with clinical interpretability. By categorizing patients into three distinct subgroups, we aimed to establish a framework with clear therapeutic implications, thereby enhancing its utility for guiding individualized treatment decisions and ensuring translational relevance.

In contrast to previous studies that predominantly concentrated on predicting the efficacy of neoadjuvant therapy (25, 26), this investigation transcends the traditional response-prediction framework by employing radiomics to discern biologically heterogeneous patient subpopulations, thus enabling the selection of those most likely to benefit from immunotherapy. This precision stratification strategy introduces an innovative paradigm for the personalization of neoadjuvant therapy in gastric cancer. Importantly, therapeutic outcomes exhibited significant variation between NICT and NACT across different radiomic score strata. Specifically, within the intermediate-score group, NICT achieved a significantly higher MPR rate compared to NACT; this result was consistently validated in both the training and validation sets, underscoring the efficacy of the radiomic signature in identifying patients with heightened sensitivity to immunotherapy, thereby informing individualized treatment planning. Subsequent univariable and multivariable logistic regression analysis corroborated that, within the intermediate-score group, NICT emerged as an independent predictor of enhanced MPR, underscoring its clinical relevance. In contrast, within the low-score group, MPR rates remained consistently low regardless of the administration of NICT or NACT, suggesting a limited response to existing standard-of-care treatments. This finding indicates a potential need for alternative therapeutic approaches, such as targeted therapies, novel immune checkpoint inhibitors, or comprehensive combination strategies (28–30). In the high-score group, the similar MPR rates observed between NICT and NACT indicate that chemotherapy alone may represent a viable option for further investigation. Consequently, future prospective studies should explore whether chemotherapy monotherapy can maintain therapeutic efficacy while reducing immune-related adverse events. However, long-term follow-up and larger-scale studies are essential to confirm the durability and safety of these regimens within this specific population.

In this study, major pathological response exhibited significant predictive value for long-term survival benefits. MPR has been extensively utilized as a surrogate endpoint in neoadjuvant therapy trials for gastric cancer, supported by numerous high-quality clinical studies (6, 31, 32). Its correlation with long-term survival outcomes is increasingly validated by empirical evidence and has garnered substantial scholarly interest (33). Establishing a stratification framework based on MPR is clinically justified and preliminarily supported by observed survival data. However, it is important to recognize that MPR cannot yet fully replace overall survival or disease-free survival as a surrogate, particularly in the context of immunotherapy; its prognostic value for long-term outcomes necessitates further validation through prospective studies.

The analysis of immune infiltration indicated potential associations between the radiomic score and the tumor immune microenvironment. Prior to the commencement of NICT, patients categorized within the intermediate-score group exhibited significantly higher baseline infiltration of dendritic cells compared to the combined high- and low-score groups. It is noteworthy that the critical role of dendritic cells in facilitating the therapeutic efficacy of PD-1/PD-L1 immune checkpoint inhibitors—primarily through their antigen-presenting functions—is extensively documented in the literature (34–36). In this study, a significant positive correlation was observed between KRT5 mRNA expression levels and CIBERSORT-estimated dendritic cell infiltration. Additionally, GSEA demonstrated that tumors with elevated KRT5 expression were significantly enriched in pathways related to antigen presentation. This suggests that the KRT5-defined tumor subtype may possess an enhanced capacity for antigen presentation, which is a fundamental requirement for effective dendritic cell–mediated antitumor immunity (37–40). It is imperative to underscore that the associations identified in this study are fundamentally indirect and observational in nature. While KRT5 emerges as a potential biomarker closely associated with dendritic cell infiltration levels, its direct involvement in immune regulation remains unsubstantiated. Rather, KRT5 may simply indicate a particular state of tumor differentiation that coincides with increased dendritic cell infiltration and heightened sensitivity to immunotherapy. As this study is grounded in retrospective observational data, the relationships identified are correlational rather than causal. critical concerns persist regarding the precise functional role of KRT5 in dendritic cells, its impact on cell maturation and antigen presentation processes, and the implications of these mechanisms for the efficacy of immunotherapy. These aspects remain insufficiently validated both experimentally and mechanistically. Consequently, the potential association between KRT5 and the tumor immune microenvironment requires urgent experimental validation. Future research should systematically explore the functional role of KRT5 in antigen presentation and its regulatory mechanisms in immunotherapy response. This can be achieved by employing molecular biology techniques, such as gene knockout and overexpression, in conjunction with *in vitro* cell experiments and *in vivo* animal models.This study is subject to several limitations. Firstly, there are imbalances in sample size and the distribution of clinical characteristics within the training and validation sets. Due to the multicenter and retrospective nature of data collection, there is heterogeneity among patients concerning treatment regimens, imaging acquisition parameters, and pathological assessment criteria. Although standardized preprocessing procedures and multivariable adjustments were implemented to mitigate these sources of variation, the possibility of residual bias cannot be entirely ruled out. Furthermore, the systemic treatment regimens administered to patients were heterogeneous, comprising different chemotherapy backbones and various immune checkpoint inhibitors. The limited sample size precluded the possibility of conducting subgroup or sensitivity analyses to fully account for potential differences in efficacy among these diverse regimens. This heterogeneity constitutes a limitation of our study. To further validate the robustness and generalizability of the proposed model, prospective multicenter studies with standardized protocols are necessary. In parallel, we acknowledge that in clinical practice, a unidimensional radiomics model is insufficient as a sole basis for decision-making in neoadjuvant immunotherapy. In addition to radiomics features, biomarkers such as the CPS, Microsatellite Instability/Mismatch Repair status, and Tumor Mutational Burden have been demonstrated to be closely associated with immunotherapy responses in gastric cancer. However, during the patient inclusion period of this study, these tests were not routinely conducted as preoperative assessments across all three participating centers, leading to high rates of missing data and preventing reliable statistical comparisons. Future prospective studies are essential for designing direct comparisons between the Radscore and other biomarkers, as well as for investigating whether their combined application can further enhance predictive accuracy. Finally, although our preliminary analysis of immune infiltration suggests potential associations between radiomic features and the tumor immune microenvironment, our current understanding of the specific biological mechanisms is still nascent. This limitation is primarily due to the restricted sample size of the immune infiltration subset and the unavoidable confounding effects arising from variations in sample types and tumor heterogeneity in bulk RNA sequencing data. While these findings offer potential biological insights into the tumor microenvironment, the current evidence is insufficient to establish causality, necessitating that these findings be strictly classified as exploratory. Future research must urgently employ spatial registration techniques that align imaging with pathology to compare imaging features with immune cell distributions within the same spatial regions, thereby facilitating the establishment of reliable macro-to-micro mappings.

In conclusion, the radiomics-based scoring system developed in this study provides a non-invasive and quantifiable method for identifying patients with locally advanced gastric cancer who are most likely to benefit from neoadjuvant therapy. This system shows promise in predicting treatment response and selecting candidates for combined immunochemotherapy. Furthermore, it may assist in identifying patients for whom immunotherapy de-escalation could be considered in future prospective studies. These findings establish a preliminary evidence-based framework that could guide individualized treatment decision-making. Future research should aim to validate the system’s applicability in larger, multicenter prospective cohorts with long-term follow-up to assess its prognostic value for survival outcomes. The integration of multi-modal imaging data, such as MRI and PET/CT, could enhance predictive accuracy and generalizability. Further investigation into the biological mechanisms underlying radiomic signatures is warranted, particularly focusing on the functional roles of key molecular biomarkers. This research should aim to elucidate their interactions with the tumor immune microenvironment and strengthen the foundation for precision therapy in gastric cancer.

## Data Availability

The original contributions presented in the study are included in the article/**Supplementary Material**. Further inquiries can be directed to the corresponding authors.

## References

[1] Sung H Ferlay J Siegel RL Laversanne M Soerjomataram I Jemal A . Global cancer statistics 2020: GLOBOCAN estimates of incidence and mortality worldwide for 36 cancers in 185 countries. CA Cancer J Clin. (2021) 71:209–49. doi: 10.3322/caac.21660 33538338

[2] Noh SH Park SR Yang HK Chung HC Chung IJ Kim SW . Adjuvant capecitabine plus oxaliplatin for gastric cancer after D2 gastrectomy (CLASSIC): 5-year follow-up of an open-label, randomised phase 3 trial. Lancet Oncol. (2014) 15:1389–96. doi: 10.1016/s1470-2045(14)70473-5 25439693

[3] Sasako M Sakuramoto S Katai H Kinoshita T Furukawa H Yamaguchi T . Five-year outcomes of a randomized phase III trial comparing adjuvant chemotherapy with S-1 versus surgery alone in stage II or III gastric cancer. J Clin Oncol. (2011) 29:4387–93. doi: 10.1200/jco.2011.36.5908 22010012

[4] Cunningham D Allum WH Stenning SP Thompson JN Van de Velde CJ Nicolson M . Perioperative chemotherapy versus surgery alone for resectable gastroesophageal cancer. N Engl J Med. (2006) 355:11–20. doi: 10.1056/nejmoa055531 16822992

[5] Zhang X Liang H Li Z Xue Y Wang Y Zhou Z . Perioperative or postoperative adjuvant oxaliplatin with S-1 versus adjuvant oxaliplatin with capecitabine in patients with locally advanced gastric or gastro-oesophageal junction adenocarcinoma undergoing D2 gastrectomy (RESOLVE): final report of a randomised, open-label, phase 3 trial. Lancet Oncol. (2025) 26:312–9. doi: 10.1016/s1470-2045(24)00676-4 39952264

[6] Lin JX Tang YH Zheng HL Ye K Cai JC Cai LS . Neoadjuvant camrelizumab and apatinib combined with chemotherapy versus chemotherapy alone for locally advanced gastric cancer: a multicenter randomized phase 2 trial. Nat Commun. (2024) 15:41. doi: 10.1038/s41467-023-44309-5 38167806 PMC10762218

[7] Livingstone E Zimmer L Hassel JC Fluck M Eigentler TK Loquai C . Adjuvant nivolumab plus ipilimumab or nivolumab alone versus placebo in patients with resected stage IV melanoma with no evidence of disease (IMMUNED): final results of a randomised, double-blind, phase 2 trial. Lancet. (2022) 400:1117–29. doi: 10.1016/s0140-6736(22)01654-3 36099927

[8] Gandhi L Rodríguez-Abreu D Gadgeel S Esteban E Felip E De Angelis F . Pembrolizumab plus chemotherapy in metastatic non-small-cell lung cancer. N Engl J Med. (2018) 378:2078–92. doi: 10.1056/nejmoa1801005 29658856

[9] Janjigian YY Shitara K Moehler M Garrido M Salman P Shen L . First-line nivolumab plus chemotherapy versus chemotherapy alone for advanced gastric, gastro-oesophageal junction, and oesophageal adenocarcinoma (CheckMate 649): a randomised, open-label, phase 3 trial. Lancet. (2021) 398:27–40. doi: 10.1016/s0140-6736(21)00797-2 34102137 PMC8436782

[10] Kang YK Chen LT Ryu MH Oh DY Oh SC Chung HC . Nivolumab plus chemotherapy versus placebo plus chemotherapy in patients with HER2-negative, untreated, unresectable advanced or recurrent gastric or gastro-oesophageal junction cancer (ATTRACTION-4): a randomised, multicentre, double-blind, placebo-controlled, phase 3 trial. Lancet Oncol. (2022) 23:234–47. doi: 10.1016/s1470-2045(21)00692-6 35030335

[11] Postow MA Sidlow R Hellmann MD . Immune-related adverse events associated with immune checkpoint blockade. N Engl J Med. (2018) 378:158–68. doi: 10.1056/nejmra1703481 29320654

[12] Ramos-Casals M Brahmer JR Callahan MK Flores-Chávez A Keegan N Khamashta MA . Immune-related adverse events of checkpoint inhibitors. Nat Rev Dis Primers. (2020) 6:38. doi: 10.7326/aitc202402200 32382051 PMC9728094

[13] Yamashita K Iwatsuki M Harada K Eto K Hiyoshi Y Ishimoto T . Prognostic impacts of the combined positive score and the tumor proportion score for programmed death ligand-1 expression by double immunohistochemical staining in patients with advanced gastric cancer. Gastric Cancer. (2020) 23:95–104. doi: 10.1007/s10120-019-00999-9 31451991

[14] Kelly RJ Zaidi AH Smith MA Omstead AN Kosovec JE Matsui D . The dynamic and transient immune microenvironment in locally advanced esophageal adenocarcinoma post chemoradiation. Ann Surg. (2018) 268:992–9. doi: 10.1097/sla.0000000000002410 28806299

[15] McLaughlin J Han G Schalper KA Carvajal-Hausdorf D Pelekanou V Rehman J . Quantitative assessment of the heterogeneity of PD-L1 expression in non-small-cell lung cancer. JAMA Oncol. (2016) 2:46–54. doi: 10.1001/jamaoncol.2015.3638 26562159 PMC4941982

[16] Ilie M Long-Mira E Bence C Butori C Lassalle S Bouhlel L . Comparative study of the PD-L1 status between surgically resected specimens and matched biopsies of NSCLC patients reveal major discordances: a potential issue for anti-PD-L1 therapeutic strategies. Ann Oncol. (2016) 27:147–53. doi: 10.1093/annonc/mdv489 26483045

[17] Ye M Huang D Zhang Q Weng W Tan C Qin G . Heterogeneous programmed death-ligand 1 expression in gastric cancer: comparison of tissue microarrays and whole sections. Cancer Cell Int. (2020) 20:186. doi: 10.1186/s12935-020-01273-0 32489322 PMC7247123

[18] Janjigian YY Ajani JA Moehler M Shen L Garrido M Gallardo C . First-line nivolumab plus chemotherapy for advanced gastric, gastroesophageal junction, and esophageal adenocarcinoma: 3-year follow-up of the phase III CheckMate 649 trial. J Clin Oncol. (2024) 42:2012–20. doi: 10.1200/jco.23.01601 38382001 PMC11185916

[19] Sun Z Zhang T Ahmad MU Zhou Z Qiu L Zhou K . Comprehensive assessment of immune context and immunotherapy response via noninvasive imaging in gastric cancer. J Clin Invest. (2024) 134(6):e175834. doi: 10.1172/jci175834 38271117 PMC10940098

[20] Lambin P Rios-Velazquez E Leijenaar R Carvalho S van Stiphout RG Granton P . Radiomics: extracting more information from medical images using advanced feature analysis. Eur J Cancer. (2012) 48:441–6. doi: 10.1016/j.ejca.2011.11.036 22257792 PMC4533986

[21] Jin W Tian Y Xuzhang W Zhu H Zou N Shen L . Non-linear modifications enhance prediction of pathological response to pre-operative PD-1 blockade in lung cancer: A longitudinal hybrid radiological model. Pharmacol Res. (2023) 198:106992. doi: 10.1016/j.phrs.2023.106992 37977237

[22] Yao X Sun C Xiong F Zhang X Cheng J Wang C . Radiomic signature-based nomogram to predict disease-free survival in stage II and III colon cancer. Eur J Radiol. (2020) 131:109205. doi: 10.1016/j.ejrad.2020.109205 32871292

[23] Zheng Y Qiu B Liu S Song R Yang X Wu L . A transformer-based deep learning model for early prediction of lymph node metastasis in locally advanced gastric cancer after neoadjuvant chemotherapy using pretreatment CT images. EClinicalMedicine. (2024) 75:102805. doi: 10.1016/j.eclinm.2024.102805 39281097 PMC11402411

[24] Guo X Chen M Zhou L Zhu L Liu S Zheng L . Predicting early recurrence in locally advanced gastric cancer after gastrectomy using CT-based deep learning model: a multicenter study. Int J Surg. (2025) 111:2089–100. doi: 10.1097/js9.0000000000002184 39715142

[25] Shen LL Zheng HL Ding FH Lu J Chen QY Xu BB . Delta computed tomography radiomics features-based nomogram predicts long-term efficacy after neoadjuvant chemotherapy in advanced gastric cancer. Radiol Med. (2023) 128:402–14. doi: 10.1007/s11547-023-01617-6 36940007

[26] Huang ZN Zhang HX Sun YQ Zhang XQ Lin YF Weng CM . Multi-cohort study in gastric cancer to develop CT-based radiomic models to predict pathological response to neoadjuvant immunotherapy. J Transl Med. (2025) 23:362. doi: 10.1186/s12967-025-06363-z 40128827 PMC11934467

[27] Chen B Khodadoust MS Liu CL Newman AM Alizadeh AA . Profiling tumor infiltrating immune cells with CIBERSORT. Methods Mol Biol. (2018) 1711:243–59. doi: 10.1007/978-1-4939-7493-1_12 29344893 PMC5895181

[28] Kawazoe A Fukuoka S Nakamura Y Kuboki Y Wakabayashi M Nomura S . Lenvatinib plus pembrolizumab in patients with advanced gastric cancer in the first-line or second-line setting (EPOC1706): an open-label, single-arm, phase 2 trial. Lancet Oncol. (2020) 21:1057–65. doi: 10.1016/s1470-2045(20)30271-0 32589866

[29] Cytryn SL Moy RH Cowzer D Shah RH Chou JF Joshi SS . First-line regorafenib with nivolumab and chemotherapy in advanced oesophageal, gastric, or gastro-oesophageal junction cancer in the USA: a single-arm, single-centre, phase 2 trial. Lancet Oncol. (2023) 24:1073–82. doi: 10.1016/s1470-2045(23)00358-3 37666264 PMC11208000

[30] Qi C Liu C Peng Z Zhang Y Wei J Qiu W . Claudin-18 isoform 2-specific CAR T-cell therapy (satri-cel) versus treatment of physician's choice for previously treated advanced gastric or gastro-oesophageal junction cancer (CT041-ST-01): a randomised, open-label, phase 2 trial. Lancet. (2025) 405:2049–60. doi: 10.1016/s0140-6736(25)00860-8 40460847

[31] Li C Tian Y Zheng Y Yuan F Shi Z Yang L . Pathologic response of phase III study: perioperative camrelizumab plus rivoceranib and chemotherapy versus chemotherapy for locally advanced gastric cancer (DRAGON IV/CAP 05). J Clin Oncol. (2025) 43:464–74. doi: 10.1200/jco.24.00795 39383487 PMC11776878

[32] Verschoor YL van de Haar J van den Berg JG van Sandick JW Kodach LL van Dieren JM . Neoadjuvant atezolizumab plus chemotherapy in gastric and gastroesophageal junction adenocarcinoma: the phase 2 PANDA trial. Nat Med. (2024) 30:519–30. doi: 10.1038/s41591-023-02758-x 38191613 PMC10878980

[33] Lin Y Sun X Li C Yang M Wu K Liu K . Three-year follow-up of neoadjuvant tislelizumab with chemotherapy in locally advanced gastric and gastroesophageal junction adenocarcinoma: revealing cancer-associated fibroblast heterogeneity corresponding to PD-1 blockade efficacy. Adv Sci (Weinh). (2026) 13(9):e08433. doi: 10.1002/advs.202508433 41327861 PMC12903965

[34] Bie N Yong T Wei Z Liang Q Zhang X Li S . Tumor-repopulating cell-derived microparticles elicit cascade amplification of chemotherapy-induced antitumor immunity to boost anti-PD-1 therapy. Signal Transduct Target Ther. (2023) 8:408. doi: 10.1038/s41392-023-01658-3 37875473 PMC10598206

[35] Chen D Jin Z Chu H Wu Y Bian Y Yuan T . DNASE1L3-expressing dendritic cells promote CD8(+) T cell function and anti-PD-(L)1 therapy efficacy by degrading neutrophil extracellular traps. Cancer Cell. (2025) 43:1758–75.e1758. doi: 10.1016/j.ccell.2025.07.014 40816293

[36] Mayoux M Roller A Pulko V Sammicheli S Chen S Sum E . Dendritic cells dictate responses to PD-L1 blockade cancer immunotherapy. Sci Transl Med. (2020) 12(534):eaav7431. doi: 10.1126/scitranslmed.aav7431 32161104

[37] Kawai T Akira S . The role of pattern-recognition receptors in innate immunity: update on Toll-like receptors. Nat Immunol. (2010) 11:373–84. doi: 10.1038/ni.1863 20404851

[38] Kureshi CT Dougan SK . Cytokines in cancer. Cancer Cell. (2025) 43:15–35. doi: 10.1016/j.ccell.2024.11.011 39672170 PMC11841838

[39] Yang K Halima A Chan TA . Antigen presentation in cancer - mechanisms and clinical implications for immunotherapy. Nat Rev Clin Oncol. (2023) 20:604–23. doi: 10.1038/s41571-023-00789-4 37328642

[40] Hu Y Zhao Q Qin Y Mei S Wang B Zhou H . CARD11 signaling regulates CD8(+) T cell tumoricidal function. Nat Immunol. (2025) 26:1113–26. doi: 10.1038/s41590-025-02192-w 40533509

