# Supplementary material for: Optimizing patient selection for neoadjuvant immunotherapy in gastric cancer via pre-treatment imaging analysis
Source: Front Immunol. 2026 Aug 5;17:1848969. doi: 10.3389/fimmu.2026.1848969 (PMC13485801; doi:10.3389/fimmu.2026.1848969)
Supplement: Supplementary file 1 [file DataSheet1.docx]

Optimizing Patient Selection for Neoadjuvant Immunotherapy in Gastric Cancer via Pre-Treatment Imaging Analysis

[Table S1.	2](#_Toc177087127)

[Table S2.	3](#_Toc177087133)

[Table S3.	4](#_Toc177087134)

[Table S4.	5](#_Toc177087135)

[Table S5..................................................................................................................................................6](#_Toc177087135)

[Figure S1.	7](#_Toc177087127)

[Figure S2.	8](#_Toc177087133)

[Figure S3.	9](#_Toc177087134)

[Figure S4.	9](#_Toc177087127)

[Figure S5.	1](#_Toc177087133)0

[Figure S6.	1](#_Toc177087134)0

[Figure S7.	1](#_Toc177087134)1

[Figure S8.	1](#_Toc177087134)1

[Figure S9.	1](#_Toc177087134)2

**Table S1** The CT protocol of the three centers

| **Parameters** | **Center 1 (FJMUUH)** | **Center 2 (ZZMH)** | **Center 3 (DPH)** |
| --- | --- | --- | --- |
| **CT version** | Somatom Perspective, Siemens Healthineers, Germany  Somatom Sensation, Siemens Healthineers, Germany | Revolution CT, GE Healthcare, USA  Discovery CT750 HD, GE Healthcare, USA | Brilliance iCT, Philips, Netherlands  Lightspeed, GE Healthcare, USA |
| **Tube voltage** | 120 kVp | 120 kVp | 120 kVp |
| **Tube current** | 100-250 mA | 120-350 mA | 100-250 mA |
| **Image matrix** | 512×512 | 512×512 | 512×512 |
| **Field of view** | 350×350 mm | 350×350 mm | 350×350 mm |
| **Reconstruction kernel** | STANDARD | STANDARD | STANDARD or B |
| **Image thickness** | 1.25 mm | 1.25 mm | 1.25 mm |

**Table S2** Univariate and multivariate logistic regression analysis examining potential predictive factors for major pathological response among patients in the combined high- and low-score groups within the training set

| Variables | Univariate Analysis | | | | Multivariate Analysis | | |
| --- | --- | --- | --- | --- | --- | --- | --- |
|  | OR | 95% CI | *p* value | OR | | 95% CI | *p* value |
| Age |  |  |  |  | |  |  |
| <65 | Ref. |  |  |  | |  |  |
| ≥65 | 0.82 | 0.50-1.36 | 0.442 | 0.67 | | 0.37-1.21 | 0.184 |
| Sex |  |  |  |  | |  |  |
| Female | Ref. |  |  |  | |  |  |
| Male | 1.07 | 0.57-2.00 | 0.832 | 1.06 | | 0.53-2.12 | 0.871 |
| BMI |  |  |  |  | |  |  |
| <25 | Ref. |  |  |  | |  |  |
| ≥25 | 1.14 | 0.62-2.08 | 0.678 | 1.15 | | 0.59-2.25 | 0.686 |
| ECOG Performance Status |  |  |  |  | |  |  |
| 0 | Ref. |  |  |  | |  |  |
| 1 | 1.23 | 0.71-2.15 | 0.460 | 1.31 | | 0.69-2.49 | 0.417 |
| 2 | 0.91 | 0.23-3.63 | 0.889 | 0.78 | | 0.17-3.60 | 0.749 |
| Treatment Period |  |  |  |  | |  |  |
| 2019 | Ref. |  |  |  | |  |  |
| 2020 | 0.95 | 0.25-3.51 | 0.934 | 1.03 | | 0.25-4.20 | 0.968 |
| 2021 | 1.29 | 0.38-4.40 | 0.688 | 1.44 | | 0.38-5.37 | 0.590 |
| 2022 | 1.82 | 0.52-6.32 | 0.347 | 3.27 | | 0.78-13.60 | 0.104 |
| 2023 | 0.70 | 0.17-2.93 | 0.625 | 1.54 | | 0.30-7.91 | 0.607 |
| CA19-9 |  |  |  |  | |  |  |
| <37 | Ref. |  |  |  | |  |  |
| ≥37 | 1.06 | 0.59-1.90 | 0.843 | 0.95 | | 0.50-1.79 | 0.875 |
| CEA |  |  |  |  | |  |  |
| <5 | Ref. |  |  |  | |  |  |
| ≥5 | 1.84 | 1.10-3.06 | 0.019 | 2.24 | | 1.28-3.93 | 0.005 |
| Clinical T stage |  |  |  |  | |  |  |
| cT3 | Ref. |  |  |  | |  |  |
| cT4 | 1.61 | 0.77-3.35 | 0.204 | 2.60 | | 1.07-6.31 | 0.035 |
| Clinical N stage |  |  |  |  | |  |  |
| cN0 | Ref. |  |  |  | |  |  |
| cN+ | 0.87 | 0.25-3.05 | 0.828 | 0.46 | | 0.11-1.95 | 0.289 |
| Tumor Location |  |  |  |  | |  |  |
| lower | Ref. |  |  |  | |  |  |
| middle | 0.58 | 0.26-1.30 | 0.189 | 0.60 | | 0.25-1.43 | 0.250 |
| upper | 1.01 | 0.54-1.89 | 0.983 | 0.91 | | 0.45-1.82 | 0.790 |
| mix | 0.76 | 0.33-1.72 | 0.509 | 0.67 | | 0.27-1.68 | 0.400 |
| Histological Grade |  |  |  |  | |  |  |
| Well/Moderately differentiated | Ref. |  |  |  | |  |  |
| Poorly/Undifferentiated | 0.96 | 0.56-1.62 | 0.867 | 0.82 | | 0.46-1.47 | 0.512 |
| Unknown | 0.70 | 0.27-1.81 | 0.458 | 0.77 | | 0.28-2.14 | 0.613 |
| Chemotherapy Regimen |  |  |  |  | |  |  |
| Doublet regimen | Ref. |  |  |  | |  |  |
| Triplet regimen | 0.58 | 0.27-1.24 | 0.159 | 0.41 | | 0.16-1.06 | 0.065 |
| Type of NAT |  |  |  |  | |  |  |
| NACT | Ref. |  |  |  | |  |  |
| NICT | 0.79 | 0.47-1.33 | 0.384 | 0.59 | | 0.33-1.07 | 0.084 |
| Abbreviations: BMI, body mass index; NAT, neoadjuvant therapy; NACT, neoadjuvant chemotherapy; NICT, neoadjuvant immunochemotherapy. | | | | | | | |

**Table S3** Univariate and multivariate logistic regression analysis examining potential predictive factors for major pathological response among patients in the intermediate-score group within the training set

| Variables | Univariate Analysis | | | Multivariate Analysis | | |
| --- | --- | --- | --- | --- | --- | --- |
|  | OR | 95% CI | *p* value | OR | 95% CI | *p* value |
| Age |  |  |  |  |  |  |
| <65 | Ref. |  |  |  |  |  |
| ≥65 | 1.30 | 0.65-2.61 | 0.460 | 1.27 | 0.54-2.98 | 0.579 |
| Sex |  |  |  |  |  |  |
| Female | Ref. |  |  |  |  |  |
| Male | 1.93 | 0.75-4.92 | 0.171 | 1.15 | 0.40-3.34 | 0.797 |
| BMI |  |  |  |  |  |  |
| <25 | Ref. |  |  |  |  |  |
| ≥25 | 0.85 | 0.35-2.07 | 0.727 | 1.06 | 0.37-3.01 | 0.913 |
| ECOG Performance Status |  |  |  |  |  |  |
| 0 | Ref. |  |  |  |  |  |
| 1 | 0.58 | 0.24-1.39 | 0.222 | 0.33 | 0.11-1.01 | 0.052 |
| 2 | 0.93 | 0.21-4.13 | 0.926 | 0.39 | 0.06-2.80 | 0.351 |
| Treatment Period |  |  |  |  |  |  |
| 2019 | Ref. |  |  |  |  |  |
| 2020 | 1.05 | 0.17-6.46 | 0.958 | 0.86 | 0.10-7.60 | 0.890 |
| 2021 | 2.04 | 0.37-11.33 | 0.415 | 1.85 | 0.24-14.14 | 0.552 |
| 2022 | 2.03 | 0.37-11.05 | 0.412 | 2.02 | 0.26-15.57 | 0.500 |
| 2023 | 1.50 | 0.18-12.46 | 0.707 | 1.42 | 0.11-19.10 | 0.791 |
| CA19-9 |  |  |  |  |  |  |
| <37 | Ref. |  |  |  |  |  |
| ≥37 | 0.64 | 0.28-1.43 | 0.274 | 0.54 | 0.20-1.49 | 0.235 |
| CEA |  |  |  |  |  |  |
| <5 | Ref. |  |  |  |  |  |
| ≥5 | 1.15 | 0.57-2.33 | 0.698 | 2.41 | 0.91-6.38 | 0.077 |
| Clinical T stage |  |  |  |  |  |  |
| cT3 | Ref. |  |  |  |  |  |
| cT4 | 0.97 | 0.36-2.65 | 0.953 | 0.99 | 0.29-3.32 | 0.986 |
| Clinical N stage |  |  |  |  |  |  |
| cN0 | Ref. |  |  |  |  |  |
| cN+ | 1.14 | 0.10-12.89 | 0.916 | 0.68 | 0.04-11.71 | 0.791 |
| Tumor Location |  |  |  |  |  |  |
| lower | Ref. |  |  |  |  |  |
| middle | 0.81 | 0.24-2.68 | 0.727 | 0.99 | 0.22-4.40 | 0.991 |
| upper | 1.03 | 0.42-2.53 | 0.950 | 0.58 | 0.18-1.86 | 0.359 |
| mix | 1.05 | 0.38-2.88 | 0.924 | 0.86 | 0.23-3.20 | 0.820 |
| Histological Grade |  |  |  |  |  |  |
| Well/Moderately differentiated | Ref. |  |  |  |  |  |
| Poorly/Undifferentiated | 0.65 | 0.31-1.37 | 0.263 | 0.49 | 0.21-1.16 | 0.104 |
| Unknown | 3.00 | 0.67-13.40 | 0.150 | 2.11 | 0.37-11.93 | 0.399 |
| Chemotherapy Regimen |  |  |  |  |  |  |
| Doublet regimen | Ref. |  |  |  |  |  |
| Triplet regimen | 0.98 | 0.31-3.09 | 0.966 | 0.61 | 0.15-2.42 | 0.483 |
| Type of NAT |  |  |  |  |  |  |
| NACT | Ref. |  |  |  |  |  |
| NICT | 2.76 | 1.35-5.64 | 0.005 | 4.34 | 1.78-10.60 | 0.001 |
| Abbreviations: BMI, body mass index; NAT, neoadjuvant therapy; NACT, neoadjuvant chemotherapy; NICT, neoadjuvant immunochemotherapy. | | | | | | |

**Table S4** Univariate and multivariate logistic regression analysis examining potential predictive factors for major pathological response among patients in the combined high- and low-score groups within the validation set

| Variables | Univariate Analysis | | | | Multivariate Analysis | | |
| --- | --- | --- | --- | --- | --- | --- | --- |
|  | OR | 95% CI | *p* value | OR | | 95% CI | *p* value |
| Age |  |  |  |  | |  |  |
| <65 | Ref. |  |  |  | |  |  |
| ≥65 | 0.95 | 0.45-2.00 | 0.893 | 0.94 | | 0.38-2.35 | 0.896 |
| Sex |  |  |  |  | |  |  |
| Female | Ref. |  |  |  | |  |  |
| Male | 1.43 | 0.62-3.29 | 0.403 | 1.56 | | 0.56-4.36 | 0.392 |
| BMI |  |  |  |  | |  |  |
| <25 | Ref. |  |  |  | |  |  |
| ≥25 | 0.32 | 0.07-1.48 | 0.145 | 0.23 | | 0.04-1.30 | 0.096 |
| ECOG Performance Status |  |  |  |  | |  |  |
| 0 | Ref. |  |  |  | |  |  |
| 1 | 1.29 | 0.58-2.89 | 0.535 | 1.25 | | 0.48-3.28 | 0.649 |
| 2 | 0.89 | 0.09-8.99 | 0.924 | 0.74 | | 0.05-10.95 | 0.826 |
| Treatment Period |  |  |  |  | |  |  |
| 2019 | Ref. |  |  |  | |  |  |
| 2020 | 1.58 | 0.44-5.76 | 0.485 | 1.91 | | 0.41-8.80 | 0.408 |
| 2021 | 1.36 | 0.41-4.53 | 0.619 | 1.19 | | 0.30-4.65 | 0.804 |
| 2022 | 1.28 | 0.43-3.84 | 0.655 | 0.86 | | 0.13-5.71 | 0.875 |
| 2023 | 1.06 | 0.17-6.68 | 0.954 | 0.40 | | 0.03-5.89 | 0.503 |
| CA19-9 |  |  |  |  | |  |  |
| <37 | Ref. |  |  |  | |  |  |
| ≥37 | 1.21 | 0.48-3.10 | 0.685 | 1.56 | | 0.52-4.73 | 0.430 |
| CEA |  |  |  |  | |  |  |
| <5 | Ref. |  |  |  | |  |  |
| ≥5 | 0.87 | 0.40-1.88 | 0.722 | 0.83 | | 0.33-2.05 | 0.680 |
| Clinical T stage |  |  |  |  | |  |  |
| cT3 | Ref. |  |  |  | |  |  |
| cT4 | 0.36 | 0.16-0.79 | 0.011 | 0.22 | | 0.08-0.60 | 0.003 |
| Clinical N stage |  |  |  |  | |  |  |
| cN0 | Ref. |  |  |  | |  |  |
| cN+ | 2.86 | 0.61-13.33 | 0.180 | 4.68 | | 0.80-27.46 | 0.088 |
| Tumor Location |  |  |  |  | |  |  |
| lower | Ref. |  |  |  | |  |  |
| middle | 0.96 | 0.37-2.52 | 0.941 | 0.94 | | 0.30-3.02 | 0.923 |
| upper | 0.84 | 0.34-2.08 | 0.712 | 1.03 | | 0.34-3.15 | 0.957 |
| mix | 0.00 | 0.00-Inf | 0.989 | 0.00 | | 0.00-Inf | 0.997 |
| Histological Grade |  |  |  |  | |  |  |
| Well/Moderately differentiated | Ref. |  |  |  | |  |  |
| Poorly/Undifferentiated | 1.05 | 0.49-2.25 | 0.908 | 1.13 | | 0.47-2.69 | 0.789 |
| Unknown | 0.00 | 0.00-Inf | 0.988 | 0.00 | | 0.00-Inf | 0.992 |
| Chemotherapy Regimen |  |  |  |  | |  |  |
| Doublet regimen | Ref. |  |  |  | |  |  |
| Triplet regimen | 0.26 | 0.03-2.10 | 0.205 | 0.24 | | 0.02-2.42 | 0.224 |
| Type of NAT |  |  |  |  | |  |  |
| NACT | Ref. |  |  |  | |  |  |
| NICT | 1.10 | 0.52-2.33 | 0.802 | 1.05 | | 0.21-5.38 | 0.952 |
| Abbreviations: BMI, body mass index; NAT, neoadjuvant therapy; NACT, neoadjuvant chemotherapy; NICT, neoadjuvant immunochemotherapy. | | | | | | | |

**Table S5** Univariate and multivariate logistic regression analysis examining potential predictive factors for major pathological response among patients in the intermediate-score group within the validation set

| Variables | Univariate Analysis | | | | Multivariate Analysis | | |
| --- | --- | --- | --- | --- | --- | --- | --- |
|  | OR | 95% CI | *p* value | OR | | 95% CI | *p* value |
| Age |  |  |  |  | |  |  |
| <65 | Ref. |  |  |  | |  |  |
| ≥65 | 1.49 | 0.59-3.79 | 0.399 | 2.33 | | 0.45-11.99 | 0.311 |
| Sex |  |  |  |  | |  |  |
| Female | Ref. |  |  |  | |  |  |
| Male | 1.80 | 0.62-5.21 | 0.277 | 1.03 | | 0.21-5.03 | 0.973 |
| BMI |  |  |  |  | |  |  |
| <25 | Ref. |  |  |  | |  |  |
| ≥25 | 1.73 | 0.36-8.35 | 0.496 | 1.88 | | 0.16-22.69 | 0.621 |
| ECOG Performance Status |  |  |  |  | |  |  |
| 0 | Ref. |  |  |  | |  |  |
| 1 | 0.80 | 0.28-2.26 | 0.673 | 0.54 | | 0.11-2.56 | 0.440 |
| 2 | 1.03 | 0.09-12.10 | 0.983 | 0.63 | | 0.01-28.81 | 0.815 |
| Treatment Period |  |  |  |  | |  |  |
| 2019 | Ref. |  |  |  | |  |  |
| 2020 | 0.80 | 0.18-3.49 | 0.766 | 0.83 | | 0.11-6.29 | 0.854 |
| 2021 | 0.73 | 0.17-3.13 | 0.669 | 0.90 | | 0.08-10.38 | 0.931 |
| 2022 | 1.09 | 0.28-4.19 | 0.899 | 0.29 | | 0.03-3.30 | 0.317 |
| 2023 | 0.91 | 0.23-3.66 | 0.893 | 0.20 | | 0.02-2.53 | 0.215 |
| CA19-9 |  |  |  |  | |  |  |
| <37 | Ref. |  |  |  | |  |  |
| ≥37 | 0.81 | 0.25-2.56 | 0.714 | 0.23 | | 0.04-1.28 | 0.094 |
| CEA |  |  |  |  | |  |  |
| <5 | Ref. |  |  |  | |  |  |
| ≥5 | 2.80 | 1.06-7.38 | 0.037 | 7.71 | | 1.45-40.85 | 0.016 |
| Clinical T stage |  |  |  |  | |  |  |
| cT3 | Ref. |  |  |  | |  |  |
| cT4 | 0.60 | 0.21-1.71 | 0.340 | 1.06 | | 0.16-6.80 | 0.952 |
| Clinical N stage |  |  |  |  | |  |  |
| cN0 | Ref. |  |  |  | |  |  |
| cN+ | 1.07 | 0.25-4.53 | 0.923 | 0.34 | | 0.03-4.28 | 0.405 |
| Tumor Location |  |  |  |  | |  |  |
| lower | Ref. |  |  |  | |  |  |
| middle | 1.69 | 0.48-6.01 | 0.416 | 7.53 | | 0.87-65.50 | 0.067 |
| upper | 0.47 | 0.13-1.67 | 0.245 | 0.74 | | 0.09-6.33 | 0.787 |
| mix | 0.00 | 0.00-Inf | 0.993 | 0.00 | | 0.00-Inf | 0.992 |
| Histological Grade |  |  |  |  | |  |  |
| Well/Moderately differentiated | Ref. |  |  |  | |  |  |
| Poorly/Undifferentiated | 0.67 | 0.26-1.76 | 0.419 | 0.43 | | 0.07-2.72 | 0.370 |
| Unknown | 0.86 | 0.07-10.66 | 0.909 | 1.20 | | 0.04-32.83 | 0.916 |
| Chemotherapy Regimen |  |  |  |  | |  |  |
| Doublet regimen | Ref. |  |  |  | |  |  |
| Triplet regimen | 0.44 | 0.09-2.22 | 0.323 | 1.70 | | 0.20-14.65 | 0.629 |
| Type of NAT |  |  |  |  | |  |  |
| NACT | Ref. |  |  |  | |  |  |
| NICT | 4.48 | 1.67-12.01 | 0.003 | 14.96 | | 1.65-135.94 | 0.016 |
| Abbreviations: BMI, body mass index; NAT, neoadjuvant therapy; NACT, neoadjuvant chemotherapy; NICT, neoadjuvant immunochemotherapy. | | | | | | | |

**Figure S1.** Enrollment process of patients in both the training and validation sets. Abbreviations: NACT, neoadjuvant chemotherapy; NICT, neoadjuvant immunochemotherapy.

Figure S2. Kaplan–Meier curves for OS and DFS stratified by MPR status in both the training and validation sets: (A) OS within the training set, (B) DFS within the training set, (C) OS within the validation set, and (D) DFS within the validation set.

**Figure S3.** An assessment of characteristic stability through the use of intra- and inter-class correlation coefficients (ICCs) for the extraction of CT radiomic features.

**Figure S4.** The application of LASSO regression: (A) illustrates the LASSO regression coefficients across different log λ values; (B) displays the partial likelihood deviance for LASSO under various log λ values; and (C) shows the LASSO coefficients for the 15 candidate features utilized in constructing a radiomic scoring system.

**Figure S5.** (A) A scatter plot depicting the distribution of radiomic scores in relation to major pathological response (MPR) status, and (B) a division of patients into tertiles based on their radiomic scores.

**Figure S6.** A comparative analysis of clinical data between the combined high- and low-score groups and the intermediate-score group within the training set.

**Figure S7.** A comparison of PD-L1 expression levels among the high-score, intermediate-score, and low-score groups in the DPH cohort.

**Figure S8.** Interactions between KRT5 and other genes within the network.

**Figure S9.** A network correlation heatmap illustrating the interrelationships among KRT5, dendritic cell activation, and radiomic features.
